# Whole exome sequencing establishes diagnosis of Charcot–Marie–Tooth 4J, 1C, and X1 subtypes

**DOI:** 10.1002/mgg3.1141

**Published:** 2020-02-05

**Authors:** Kleita Michaelidou, Ioannis Tsiverdis, Sophia Erimaki, Dimitra Papadimitriou, Georgios Amoiridis, Alexandros Papadimitriou, Panayiotis Mitsias, Ioannis Zaganas

**Affiliations:** ^1^ Neurogenetics Laboratory Medical School University of Crete Heraklion, Crete Greece; ^2^ Neurology Department University Hospital of Crete Heraklion, Crete Greece; ^3^ Neurophysiology Unit University Hospital of Crete Heraklion, Crete Greece; ^4^ Henry Dunant Hospital Center and Biomedical Research Foundation Academy Athens Greece; ^5^ Department of Neurology Henry Ford Hospital/Wayne State University Detroit MI USA

**Keywords:** Charcot–Marie–Tooth disease, genetics, inherited polyneuropathy, whole exome sequencing

## Abstract

**Background:**

Charcot–Marie–Tooth (CMT) hereditary polyneuropathies pose a diagnostic challenge. Our aim here is to describe CMT patients diagnosed by whole exome sequencing (WES) following years of fruitless testing.

**Methods/Results:**

Three patients with polyneuropathy suspected to be genetic in origin, but not harboring *PMP22* gene deletion/duplication, were offered WES. The first patient, a 66‐year‐old man, had been suffering from progressive weakness and atrophies in the lower and upper extremities for 20 years. Due to ambiguous electrophysiological findings, immune therapies were administered to no avail. Twelve years after *PMP22* deletion/duplication testing, WES revealed two pathogenic variants in the *FIG4* gene (p.Ile41Thr and p.Phe598fs, respectively), as a cause of CMT 4J. The second patient, a 19‐year‐old man, had been suffering from hearing and gait impairment since at least his infancy, and recently presented with weakness and dystonia of the lower extremities. In this patient, WES identified the p.Leu122Val *LITAF* gene variant in heterozygous state, suggesting the diagnosis of CMT 1C, several years after initial genetic analyses. The third patient, a 44‐year‐old man, presented with progressive weakness and atrophies of the lower and upper extremities since the age of 17 years old. In this patient, WES identified the hemizygous p.Arg164Gln pathogenic variant in the *GJB1* gene, establishing the diagnosis of CMT X1, 8 years after testing for *PMP22* deletion/duplication.

**Conclusion:**

Novel diagnostic techniques, such as WES, offer the possibility to decipher the cause of CMT subtypes, ending the diagnostic Odyssey of the patients and sparing them from unnecessary and potentially harmful treatments.

## Graphical abstract

Here we describe three cases of Charcot–Marie–Tooth hereditary polyneuropathy (subtypes 4J, 1C, and X1, respectively) that we have diagnosed using whole exome sequencing (WES) after several years of nondiagnostic genetic and nongenetic tests. These cases demonstrate the advantages of a simplified strategy in diagnosing hereditary peripheral neuropathies, namely proceeding directly from *PMP22* deletion/duplication analysis to WES for the diagnostic evaluation of these patients.

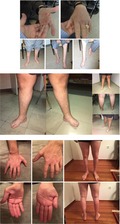

## 1 INTRODUCTION

Polyneuropathies are usually associated with significant morbidity and substantial health care costs, especially when their cause remains elusive despite multiple nondiagnostic tests. There are numerous causes of polyneuropathy, including autoimmune and metabolic disorders, toxins, infections, nutritional deficiencies, etc. (Visser, Notermans, Linssen, van den Berg, & Vrancken, 2015). However, a significant proportion of polyneuropathies are due to genetic defects (Eggermann et al., 2018), with the most common of the hereditary neuropathies being the Charcot–Marie–Tooth (CMT) group of disorders (Vaeth, Vaeth, Andersen, Christensen, & Jensen, 2017). CMT neuropathies are often a cause of significant functional impairment and even reduced life expectancy (Vaeth et al., 2017).

Charcot–Marie–Tooth disease comprises several different entities characterized by heterogeneous phenotypic features and caused by pathogenic variants in more than 80 genes (Rossor, Polke, Houlden, & Reilly, 2013). Among these, the most common genetic defect leading to a CMT phenotype is *PMP22* gene duplication (Rossor et al., 2013). However, the great majority of CMT cases are caused by pathogenic variants in multiple other genes (Hoyle, Isfort, Roggenbuck, & Arnold, 2015), hindering targeted genetic diagnosis. This diagnostic obstacle has been overcome in recent years by next generation sequencing techniques, including whole exome sequencing (WES), that have revolutionized the way we approach the diagnosis of inherited polyneuropathies (Dohrn et al., 2017; Hartley et al., 2018; Rossor et al., 2013).

Here we describe three cases with inherited polyneuropathy that have been diagnosed by us using a WES approach after several years of nondiagnostic genetic and nongenetic tests. These cases demonstrate the usefulness and practicality of a simplified approach in diagnosing hereditary peripheral neuropathies, namely proceeding directly from *PMP22* deletion/duplication analysis to WES for the diagnostic evaluation of these patients.

## 2 MATERIALS AND METHODS

### 2.1 Study subjects

This case series includes three unrelated patients with suspected hereditary peripheral neuropathy, based on clinical presentation, electrophysiological studies, and the absence of known acquired causes of polyneuropathy (Table 1). All patients had tested negative for deletion/duplication of the *PMP22* gene in the past. Informed consent for performing clinical WES was obtained from the patients and/or their next of kin or legal guardian. The study protocol followed the ethical guidelines of the World Medical Association Declaration of Helsinki (version 2008), and was also approved by the Institutional Review Board of the University Hospital of Heraklion, Crete, Greece.

**Table 1:** CMT cases diagnosed by WES in Neurology Laboratory, University of Crete

Patient number	#1	#2	#3
Sex	Male	Male	Male
Current age (years)	66	19	44
Age at symptom onset (years)	50	2	17
History/clinical features	Gait difficulties, atrophies and weakness of the lower and later the upper extremities	Gait difficulties, atrophies and weakness of the lower and later the upper extremities	Gait difficulties, atrophies and weakness of the lower and later the upper extremities
Additional clinical features	Parkinsonism	Hearing loss, dystonia	—
NCS/EMG features overview^a^	Demyelinating type sensory motor neuropathy, resembling CIDP	Demyelinating type sensory motor neuropathy	Demyelinating type sensory motor neuropathy
Brain imaging features	Normal MRI findings, abnormal DATSCAN	Normal MRI findings	Few small foci of abnormal signal intensity at subcortical white matter of the cerebral hemispheres on MRI
Lumbar puncture results	0 nucleated cells/μl, 85 mg/dl protein	0 nucleated cells/μl, 46 mg/dl protein	Not performed
Additional diagnostic investigations	Nerve Ultrasound	—	—
Medical treatments tried	IV Ig, steroids	Botulinum toxin, levodopa (for dystonia)	—
Family history	Yes (brother with possible ALS)	Yes	No
Genetic tests performed before WES	*PMP22* duplication	*PMP22* duplication, *CJB1* exon 2 sequencing	*PMP22* duplication
Age at first diagnostic test—*PMP22* duplication (y)	55	13	36
Age at diagnosis with WES (y)	63	18	44
Gene (OMIM number)	*FIG4* (609390)	*LITAF* (603795)	*GJB1* (304040)
Genetic variants (rs)	rs121908287, ‐	rs104894522,	c.491G>A(rs1241595912)
HGVS nomenclature	NM_014845.5: c.122T>C; NP_055660.1: p.I41T/NM_014845.5: c.1795delC; NP_055660.1: p.H599fs*24	NM_004862.3: c.364C>G; NP_004853.2: p.L122V	NM_000166.5: c.491G>A; NP_000157.1: p.R164Q
Variants (zygosity)	p.Ile41Thr (heterozygous)/p.His599Ilefs*24 (heterozygous)	p.Leu122Val (heterozygous)	p.Arg164Gln (hemizygous)
Functional consequence (Ingenuity Classification)	Pathogenic/pathogenic	Uncertain significance	Likely pathogenic
Functional consequence (ClinVar Classification)	Pathogenic/not reported	Pathogenic	Pathogenic
CADD score	26.1/27.7	25.5	28.6
Mode of inheritance	Autosomal recessive	Autosomal dominant	X‐linked dominant
CMT type (OMIM number)	4J (611228)	1C (601098)	X1 (302800)

Abbreviations: ALS, amyotrophic lateral sclerosis; CADD, combined annotation‐dependent depletion (Rentzsch et al., 2018); CIDP, chronic inflammatory demyelinating polyneuropathy; CMT, Charcot–Marie–Tooth; EMG, electromyography; HGVS, Human Genome Variation Society; NCS, nerve conduction studies; WES, whole exome sequencing.

a For details, see Table 2 and supplementary material.

### 2.2 Blood sampling and DNA extraction

From all participants, peripheral blood (approximately 5 ml) was collected in ethylenediaminetetraacetic acid tubes and stored at −80°C until use. DNA was extracted from 1 ml of whole blood, using the QIAamp DNA blood midi kit (Qiagen) following the manufacturer's centrifugation‐based protocol. DNA concentration and purity were determined spectrophotometrically by the absorbance measurement at 260 and 280 nm, and agarose electrophoresis was performed for verification of DNA quality.

### 2.3 Whole exome sequencing

Whole exome sequencing and bioinformatics analysis were performed in a CLIA‐certified laboratory (Otogenetics Corporation). Exome library preparation was performed using the Agilent V5 (51Mb) SureSelect Target Enrichment System. Exon‐enriched DNA libraries were sequenced on a HiSeq 2500 (Illumina) platform using paired end reads of 100–125 bp with an estimated average coverage of 50X. The data were then processed using the DNA‐Nexus platform, consisting, for each patient, of read alignment to the human reference genome hg19/GRCh37, removal of PCR duplicates using Picard, indel realignment and base quality score recalibration, variant calling, and quality evaluation using the Genome Analysis ToolKit version 3.6. Analysis of sequencing data revealed uniform coverage and high read depths in all samples. On average, the percentage of nucleotides with at least 50x coverage was more than 60%, and the average depth of coverage per interval was over 60.

### 2.4 Data analysis

Whole exome sequencing data analysis was performed at the Neurology Laboratory, University of Crete using the Ingenuity Variant Analysis (IVA) software (Qiagen). The VCF format file was analyzed via IVA, using a comprehensive stepwise filtering strategy, to generate a list of disease‐associated variants. We excluded variants with minor allele frequency >1% based on databases such as Exome Aggregation Consortium (ExAC) dataset and focused on exonic variants which produced a missense, nonsense, frameshift, or splicing change. We also kept all genetic variants that are listed in the Human Gene Mutation Database (HGMD^®^) and showed potential pathogenicity. The functional consequences (deleterious, damaging, or neutral) of identified variants on encoded proteins were also assessed using the VarSome database (https://varsome.com) that compiles prediction scores from several prediction algorithms including SIFT, PROVEAN, MutationTaster, PhyloP, FATHMM, and MetaSVM. Final interpretation of the pathogenicity of the identified variants was performed manually taking into account data available in public databases and published in the literature.

Variants related to patient phenotypes were confirmed by Sanger sequencing for the patients, parents, and affected relatives, if applicable.

## 3 RESULTS—CASE DESCRIPTIONS

The first patient (#1), a 66‐year‐old man, was suffering from progressive weakness and muscular atrophy, initially of the lower and subsequently of the upper extremities, for about 20 years. Specifically, 20 years before final diagnosis, the patient observed “atrophy” of the calves bilaterally, reportedly without motor impairment. Five years later, he noticed weakness of the distal parts of the lower extremities. When first seen by a neurologist, the diagnosis of demyelinating sensory motor neuropathy was considered likely and testing for *PMP22* deletion/duplication was negative. Ten years ago, the patient started experiencing weakness of the hands, initially on the right.

The patient reported a biopsy from the calf (possibly muscle biopsy) due to “muscle paresis,” at about the age of 20 years, but no records were available from this testing. He had a long‐standing history of hypertension, had suffered a myocardial infarction 20 years prior to the current evaluation (undergoing angioplasty of the coronary vessels twice), had presented in the past with recurrent trigeminal neuralgia, and was a heavy smoker and a social drinker. His current medications included aspirin, diltiazem, enalapril, atorvastatin, and clopidogrel.

The patient's brother had died at the age of 64 years due to an unknown neurological disorder that rendered him quadriplegic and in need for tracheostomy. Antemortem, he had been diagnosed at various times during his disease course as “possible multiple system atrophy,” “Stiff‐person syndrome,” or “spastic paraparesis.” The patient's father had died at a rather old age from stroke.

**Figure 1:**
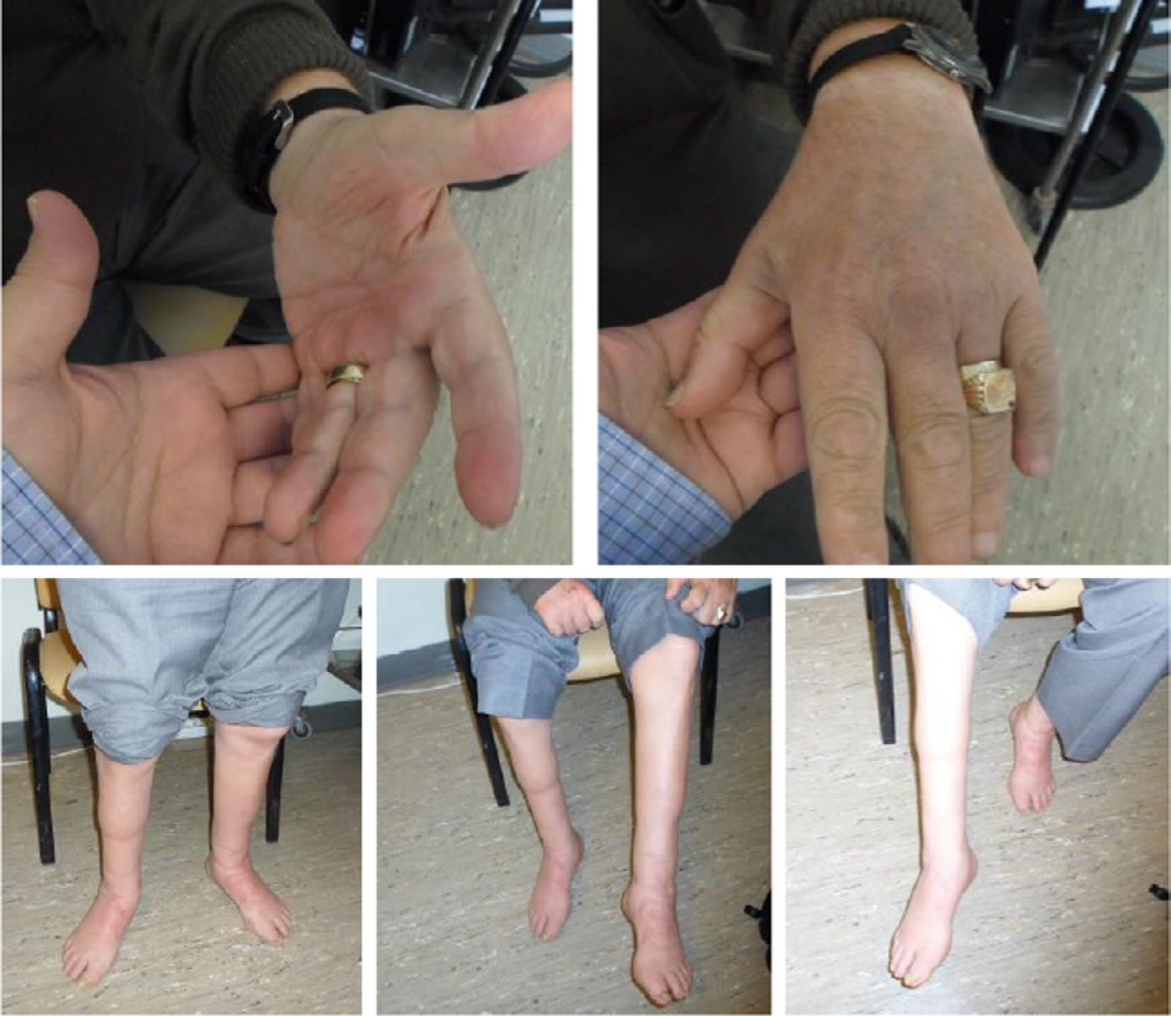
(Patient #1). Photographs of the patient's hands and lower extremities showing marked muscle atrophies. The patient was harboring the p.Ile41Thr and the p.His599Ilefs*24 pathogenic variants in the *FIG4* gene in cis heterozygous state and was diagnosed with autosomal recessive CMT 4J. See also Video S1. CMT, Charcot–Marie–Tooth

Neurological examination revealed that the patient had intact higher cognitive functions. Examination of the cranial nerves did not reveal any abnormal findings, except for anisocoria (3 mm on the right, 2 mm on the left), with normal direct and consensual responses to light and accommodation. There was marked atrophy of the hand interosseous muscles and the thenar eminences, as well as of the calves and feet bilaterally (Figure 1). On motor testing, there was weakness of the abduction and adduction of the fingers bilaterally (4/5 in the MRC scale). In the lower extremities, there was weakness in the plantar (4/5) and dorsal (3/5), flexion and inversion (4/5), and eversion (4/5) of the feet bilaterally. No significant weakness was found in the proximal muscles of the upper and lower extremities. Tendon reflexes were absent in all four extremities and plantar responses were not elicited bilaterally. There was no ataxia on finger–nose or heel–shin testing. On sensory examination, there was hypoalgesia and reduced temperature sensation in a stocking‐glove distribution. There were also mild deficits in joint position sense peripherally in all four extremities, more pronounced in the lower extremities. Pallesthesia was abolished in both feet and severely reduced in both hands (3/8 in the fingers). The patient could not walk on heels or on toes, and had a steppage type of gait bilaterally.

Motor nerve conduction studies of our patient in 2009, at the age of 57 years, showed the absence of compound muscle action potentials (CMAP's) on the peroneal and tibial nerve on the tested left side. In the upper extremities, there was increased distal latency (3.5 m in the ulnar nerve to 5.7 ms in the median nerve), and decreased motor conduction velocity (in the range of 18–32 m/s), of the median and ulnar nerve bilaterally. On sensory testing, there were no sensory nerve action potentials recorded in the sural, median, and ulnar nerve on the tested left side. On electromyography (EMG), there was spontaneous activity and neuropathic recruitment pattern in the anterior tibial muscles bilaterally. A second neurophysiological examination in 2014 (Table 2, Table S1) showed findings possibly consistent with chronic inflammatory demyelinating polyneuropathy (CIDP), namely increased distal latencies, decreased conduction velocities, the absence or prolonged latencies of F‐waves with A‐waves, and proximal conduction blocks. EMG revealed fibrillation potentials and positive sharp waves in the tibialis anterior muscles on both sides, along with a neuropathic pattern of motor unit recruitment. The same pattern of recruitment was observed in the gastrocnemius, rectus femoris, and extensor digitorum communis muscles. Reduced motor unit recruitment was observed in the left deltoid muscle.

**Table 2:** Electrophysiological testing results of CMT cases diagnosed by WES in Neurology Laboratory, University of Crete^a^

Nerve stimulated	Recording site	Stimulation site	Patient #1		Patient #2		Patient #3	
			Distal latency (ms)	Conduction velocity (m/s)	Distal latency (ms)	Conduction velocity (m/s)	Distal latency (ms)	Conduction velocity (m/s)
Tibial (m) L	AH	Ankle	NR	NR	NR	NR	**8.0**	**—**
		Popliteal fossa					**—**	**33**
Tibial (m) R	AH	Ankle	NR	NR	NR	NR	**8.8**	**—**
		Popliteal fossa					**—**	**36**
Median (m) L	APB	Wrist	**6.1**	**—**	**4.8**	**—**	**4.7**	**—**
		Antecubital fossa	**—**	**25.0**	**—**	**31.8**	**—**	**37**
Ulnar (m) L	ADM	Wrist	**4.4**	**—**	**3.5**	**—**		
		Below elbow	**—**	**20.2**	—	**26.4**	3.7	**—**
		Above elbow	—	**22.0**	—	**25.0**	—	**37**
Median (m) R	APB	Wrist	**8.3**	**—**	**4.4**	**—**	**4.6**	**—**
		Antecubital fossa	**—**	**17.4**	**—**	**30.7**	**—**	**38**
Ulnar (m) R	ADM	Wrist	**6.0**	**—**	2.5	—	**5.0**	**—**
		Below elbow	**—**	**21.8**	—	**25.0**	—	**33**
		Above elbow	**—**	**NR**	—	**23.8**	—	NR
Median (s) L	Wrist	Index finger	NR	NR	NR	**41.2**	NR	NR
Ulnar (s) L	Wrist	Little finger	NR	NR	NR	**38.0**	NR	NR

Note

Abnormal values are shown in bold.

Abbreviations: ADM, abductor digiti minimi; AH, abductor hallucis; APB, abductor pollicis brevis; CMT, Charcot–Marie–Tooth; NR, not recorded; WES, whole exome sequencing.

a More extended neurophysiological testing results are shown in the supplementary material.

*Nerve ultrasound* study showed increase in the size of the median and ulnar nerves in the middle portion of both the arm and the forearm, and not in the carpal tunnel or the elbow. Both the electrophysiological and nerve ultrasound findings, the results from a lumbar puncture performed at the time (that showed increased protein and no nucleated cells), and the negative testing for *PMP22* deletion/duplication (see below), were interpreted as possibly indicative of CIDP. For this reason, intravenous immune globulin was administered repeatedly. However, despite an initial subjective improvement, no objective changes indicating amelioration were noted on neurological examination. On the contrary, the patient's signs and symptoms (weakness, atrophies and gait difficulties) progressively worsened and the patient had to use a cane to walk.

In addition, the patient lately developed parkinsonism, gait unsteadiness, and frequent falls, that were nonresponsive to antiparkinsonian medications (Video S1). Brain single‐photon emission computed tomography for detecting dopamine transporters (DaTSCAN) showed markedly reduced (essentially abolished) uptake of the radiotracer (ioflupane iodine‐123) in the basal ganglia bilaterally (only diffuse background uptake detected).

Given the protracted course of the neurological syndrome, the clinical picture, the possible positive family history, and the negative testing for *PMP22* deletion/duplication (to rule out CMT 1A), we offered WES analysis to the patient. This WES analysis revealed the presence of two heterozygous pathogenic variants in the *FIG4* gene (OMIM 609390), namely the known pathogenic variant c.122T>C (p.Ile41Thr), and the novel frameshift variant c.1795delC (p.His599Ilefs*24), respectively. These variants were verified by Sanger sequencing and we established that they were in different alleles by testing the patient's unaffected daughter, who harbored only the His599Ilefs*24 variant. These results were diagnostic of CMT 4J.

The second patient (#2), a 19‐year‐old man, was evaluated by us due to gait difficulties since his infancy and, lately, dystonia of the lower extremities. The patient started walking independently at the age of 2 years, but he has been having gait difficulties since then. Recently, he manifested dystonia in the lower extremities, which further hindered his ability to walk. He had undergone electrophysiological testing at a younger age, with results compatible with chronic hereditary polyneuropathy with demyelinating features. As a therapeutic trial for his dystonia, the patient received levodopa and botulinum toxin with only mild improvement.

The remaining of the medical history was notable for severe hearing impairment and mild learning difficulties at his school. Concerning family history, the patient's mother (not available for clinical examination or genetic testing) had reportedly shown similar gait impairment since her young age.

**Figure 2:**
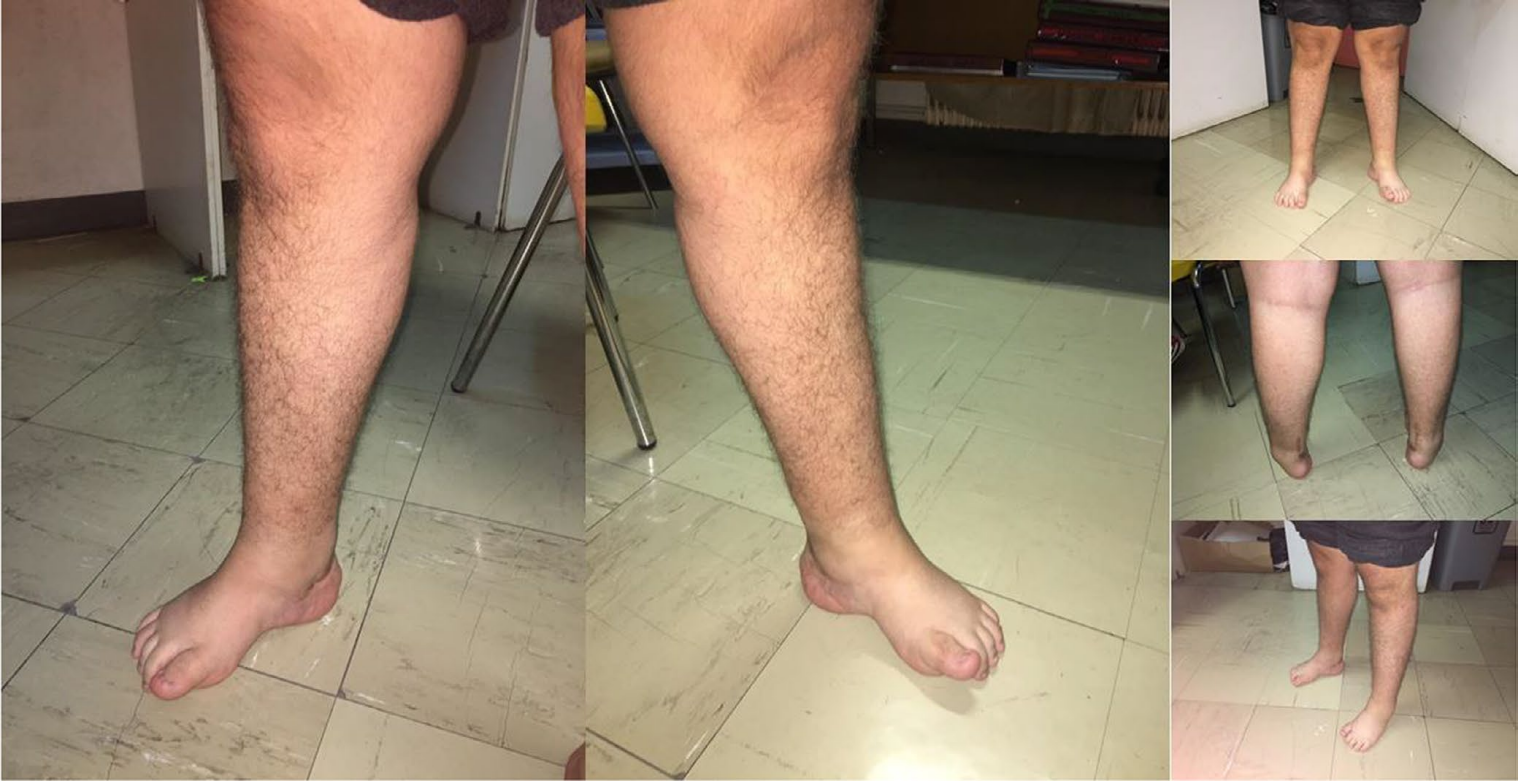
(Patient #2). Photographs of the patient's lower extremities showing marked distal atrophies. The patient was diagnosed with autosomal dominant CMT 1C, due to a heterozygous pathogenic variant (p.Leu122Val) in the *LITAF* gene. See also Video S2. CMT, Charcot–Marie–Tooth

On physical examination, the patient had a minor ulceration on the surface of the tongue. On feet inspection, we noted pes cavus and atrophies bilaterally (Figure 2), and an ingrown nail of the big toe on the right. On neurological examination, the patient was well‐oriented in time, place, and person, and his short‐term memory was not impaired. However, he showed mild deficits in other higher cognitive functions, specifically in visuospatial abilities, repetition, and backward digit span (MoCA test score 27/30). He had a high‐pitched voice but no dysarthria. Visual field testing on confrontation was intact. The light pupillary reflex was normal; however, there was mild anisocoria (3 mm on the right, 2.3 mm on the left). Ocular movements were full, but there was alternating horizontal nystagmus that was sustained, both on the left and the right gaze position. Sensation on the face was normal, as normal were frontal wrinkling, lid closure, and the glabellar reflex. On showing the teeth, there was mild asymmetry, with possible effacement of the nasolabial fold on the right. There was decreased auditory acuity bilaterally. The uvula was in mid position and the mobility of the tongue was normal.

On examination of the motor system, there was decreased muscle strength peripherally, graded according to the BMRC scale 4/5 and 3/5 on the upper and lower limbs, respectively. Deep tendon reflexes were decreased in the upper extremities, whereas in the lower extremities they were abolished. Hoffman and Babinski signs were not present. Sensory examination showed no significant deficits in pain and thermal perception, light, and discriminative touch or joint position sense. There was however a deficit in the vibration perception, more pronounced in the toes (fingers 7/8, big toe 4/8). Cerebellar testing did not show ataxia or dysdiadochokinesia. There was no tremor noted (at rest, positional or intention). Romberg testing showed a postural sway with eyes closed. The patient had difficulty in walking, with his gait being wide (Video S2). He was unable to perform tandem walking and had also difficulty walking on heels and toes. The patient assumed a dystonic posture of the big toe together with tip‐toe walking. He received a short‐term trial with L‐dopa, to exclude dopa‐responsive dystonia, with no improvement.

*Neurophysiological testing* (Table 2, Table S2) showed findings of demyelinating sensory motor polyneuropathy, with mildly decreased conduction velocities, significant prolongation of F‐wave latencies, and evidence of proximal conduction block. There was also evidence for an involvement of cranial nerves, as manifested by the increased latency of R1 response of the blink reflex (14.8 ms on both sides, normal values <13.0 ms). The EMG records showed no signs of acute denervation (positive sharp waves or fibrillation potentials). However, a distinct pattern of chronic reinnervation with decreased recruitment of large motor units of increased duration was observed in the tibialis anterior and first dorsal interosseus muscle on the left side. Compared to the muscles examined in the left lower extremity, the right peroneus brevis muscle showed a more pronounced neuropathic type of involvement.

Cerebrospinal fluid (CSF) analysis showed zero nucleated and 22 red blood cells per μl. CSF levels of glucose, protein, and lactate dehydrogenase were 62 mg/dl, 46.4 mg/dl, and 16 mg/dl, respectively. There were oligoclonal bands unique to the CSF and the IgG Index was within normal range. Serum testing showed decreased free testosterone (6.29 pg/ml, normal 9–41 pg/ml) and growth hormone (0.059 ng/ml, normal 0.06–5 ng/ml), increased adrenocorticotropic hormone‐ACTH (62.9 pg/ml, normal 5–46 pg/ml), no response to the gonadotropin‐releasing hormone (GnRH) test, borderline results in the oral glucose tolerance test and increased α2 globulins (13.5%, normal 6.9%–12.9%) but without the presence of monoclonal protein. Urine testing showed increased 24‐hr urine protein (125 mg/L, normal 0–100 mg/L) and decreased random sample urine chloride (87 mEq/L, normal 110–250 mEq/L). Brain MRI revealed no abnormalities.

Genetic testing 5 years earlier had detected neither *PMP22* duplication (for CMT1A) nor a pathogenic mutation at exon 2 of connexin‐32 (for X‐linked hereditary motor and sensory polyneuropathy, CMT‐X). WES revealed the presence of the pathogenic missense variant c.364C>G (p.Leu122Val) in the *LITAF* gene (also known as *SIMPLE*, OMIM 603795) in heterozygous state, suggesting the diagnosis of CMT type 1C. Parental samples were not available for testing.

The third patient (#3), a 44‐year‐old man, presented with progressively worsening weakness and atrophies of the upper and lower extremities. Specifically, at the age of 17 years, due to muscle atrophy of the lower extremities, he consulted a neurologist, and the clinical and electrophysiological diagnosis of CMT disease was made. Since then he has been experiencing a progressively worsening weakness of the lower extremities, and since the age of 30 years additional weakness of the upper extremities. His past medical history was unremarkable except for an episode of hematuria of unknown cause in childhood.

**Figure 3:**
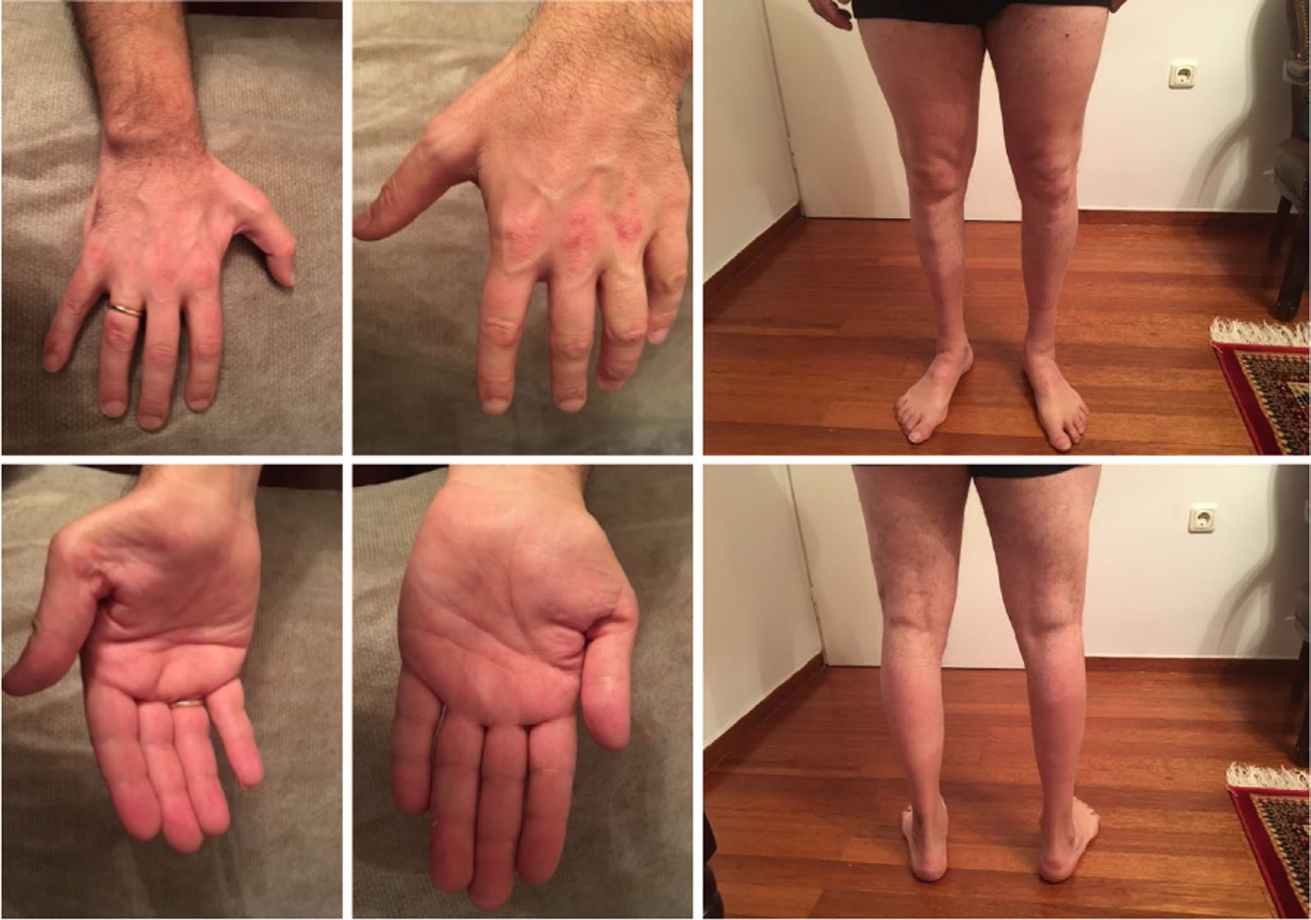
(Patient #3). Photographs of the patient's upper and lower extremities showing marked distal atrophies. The patient is affected with X‐linked CMT X1, due to the p.Arg164Gln variant in the *GJB1* gene. See also Video S3. CMT, Charcot–Marie–Tooth

On physical examination, there were marked atrophy of the calves and feet, and pes cavus bilaterally (Figure 3). On the hands, moderate atrophy of the thenar eminence and of the dorsal interosseous muscles and finger clubbing bilaterally were noted (Figure 3). Muscle strength, graded according to the MRC scale, was 3+/5 centrally in all four extremities, 3+/5 in wrist extensors, 3/5 in the plantar flexors of the foot, and 2/5 in the dorsiflexors of the foot bilaterally. The patient had gait impairment showing steppage type gait and complete inability to walk on his heels (Video S3). Tendon reflexes were absent in all four extremities and plantar responses were flexor bilaterally. Abdominal reflexes were abolished. Romberg sign was positive, with a tendency for the patient to fall to the left; this could not be accounted for by the absence of significant sensory disturbances but could be attributed to subclinical involvement of the sensory tracts. Also, there was no dysmetria when performing the finger–nose and heel–shin tests. Higher cognitive function and cranial nerve testing showed no significant abnormalities.

Electrophysiological testing many years earlier (Table 2, Table S3), at the age of 18 years, showed moderately decreased median and ulnar nerve motor conduction velocities (in the range of 33–38 m/s), with increased sensory and motor latencies in both nerves. In the lower extremities, it was not possible to elicit CMAPs of the peroneal nerves, whereas there was mild reduction in the motor conduction velocity of both tibial nerves (in the range of 33–36 m/s). EMG testing showed evidence of acute denervation (positive sharp waves and fibrillation potentials). There was also a distinct pattern of chronic reinnervation with decreased recruitment of large motor units with increased duration along with polyphasia in most of the tested muscles (Table S3).

Laboratory testing, including CBC, serum biochemistry, thyroid function testing, virology screening, and screening for autoimmune disorders, failed to reveal abnormal findings. Brain MRI scan showed a few small foci of abnormal (high on T2 sequences) signal intensity of uncertain clinical significance in the subcortical white matter, given that the patient had no signs or symptoms suggestive of CNS involvement. Incidentally, a venous angioma at the lower left basal ganglia was also found.

The patient had been tested for *PMP22* deletion/duplication about 10 years earlier, in order to rule out CMT 1A or Hereditary Neuropathy with Liability to Pressure Palsies, and the results were negative. WES identified the hemizygous c.491G>A (p.Arg164Gln) known pathogenic variant in gap junction protein β‐1 gene (*GJB1*; OMIM 304040), establishing the diagnosis of CMT X1.

## 4 DISCUSSION

In three male patients suffering from polyneuropathy, three different types of CMT were identified using WES analysis. Specifically, pathogenic variants were found in the *FIG4*, *LITAF,* and *GJB1* genes as a cause for CMT 4J, CMT 1C, and CMT X1, respectively. In all three patients, a long period of time had elapsed, and significant effort was spent, between their initial diagnostic investigations and the final diagnosis.

Patient #1 was suffering from CMT 4J due to two pathogenic mutations in the *FIG4* gene (p.Ile41Thr and p.His599Ilefs*24, respectively). The p.Ile41Thr *FIG4* variant is the most common variant described in patients with CMT 4J (Chow et al., 2007; Cottenie et al., 2013; Gentil et al., 2017; Menezes et al., 2014; Orengo, Khemani, Day, Li, & Siskind, 2018; Zhang et al., 2008), with a population frequency of 0.001 by screening 5,769 Northern European controls (Nicholson et al., 2011). A functional study by Lenk et al. (2011), showed that the p.Ile41Thr amino acid substitution results in an unstable protein in vivo. Concerning the p.His599Ilefs*24 *FIG4* variant, it has not been previously described in the literature. However, it is predicted to disrupt the structure and function of the protein by altering the reading frame of the gene, and leading to premature termination. Thus, the protein encoded by the mutated *FIG4* gene is both truncated and with altered sequence in its C‐terminal part. This frameshift variant is predicted to be pathogenic since loss‐of‐function variants in the *FIG4* gene are a known mechanism for the development of CMT4J. In addition, the high Combined Annotation Dependent Depletion (CADD) (Rentzsch, Witten, Cooper, Shendure, & Kircher, 2018) score (27.7) lends support to the pathogenicity of this variant (Table 1). In several studies, the majority of or all the patients with CMT 4J harbor the p.Ile41Thr *FIG4* ancestral mutation *in trans* with a frameshift variant, a combination occurring in our patient (Chow et al., 2007; Nicholson et al., 2011).

CMT 4J is a very rare form of CMT disease, being found in 0.3% of 3,216 CMT patients that tested positive for a genetic cause in a recent analysis of over 17,000 individuals with neuropathy (DiVincenzo et al., 2014). It is characterized by autosomal recessive mode of inheritance, variable disease onset, and phenotypic characteristics and caused by biallelic pathogenic variants in the *FIG4* gene (Chow et al., 2007; Gentil et al., 2017; Lenk et al., 2011; Menezes et al., 2014; Nicholson et al., 2011; Pinto, Oliveira, & Souza, 2015; Zhang et al., 2008). Besides causing the phenotype defined as CMT 4J, mutations in FIG4 have been associated with other neurological phenotypes, including the Yunis‐Varon syndrome (an autosomal recessive developmental disorder) (Campeau et al., 2013; Nakajima et al., 2013; Yunis & Varón, 1980), amyotrophic lateral sclerosis type 11 (inherited as an autosomal dominant trait) (Bertolin et al., 2018; Chow et al., 2009; Dols‐Icardo et al., 2018; Osmanovic et al., 2017), and autosomal recessive familial epilepsy with polymicrogyria (Baulac et al., 2014).

The lipid phosphatase encoded by the *FIG4* gene is a phosphoinositide 5‐phosphatase involved in the metabolism and availability of phosphatidylinositol‐3,5‐bisphosphate PI(3,5)P2 (Martyn & Li, 2013). There is evidence that a *FIG4* dependent signaling pathway is essential for lysosomal function, perhaps independently of its phosphatase function (Bharadwaj, Cunningham, Zhang, & Lloyd, 2016), raising the possibility that the *FIG4* associated phenotypes are another type of lysosomal storage disorder (Martyn & Li, 2013). In addition, it has been found that *FIG4* has a protective role in the adult nervous system, especially in the periphery (Mironova et al., 2018).

On retrospective analysis, several of the atypical features of the clinical course of patient #1 can be explained by the variability in the phenotype of CMT 4J. For example, it has been previously described that the pathophysiology of CMT 4J resembles that of acquired polyneuropathies (Hu et al., 2018), and that some patients with pathogenic variants in *FIG4* have been initially misdiagnosed as CIDP (due to clinical, electrophysiological, and imaging features common in both CIDP and CMT 4J, such as asymmetric involvement, conduction blocks, and temporal dispersion). These patients had received immune suppressive treatments, including intravenous immune globulin and steroids, with no improvement, as was the case in our patient (Cottenie et al., 2013; Nicholson et al., 2011). This lack of improvement argues against the possibility that CMT 4J in our patient coexists with an acquired disorder, such as CIDP, an occurrence that has been occasionally described (Rajabally, Adams, Latour, & Attarian, 2016). Furthermore, it has already been shown that patients with CMT 4J can develop parkinsonism and frequent falls in their disease course, again as occurring in our patient (Nicholson et al., 2011; Orengo et al., 2018). Finally, the clinical course of the patient's deceased brother and the possible diagnoses of multiple system atrophy or amyotrophic lateral sclerosis, available only through descriptions by the patient's family, could be compatible with the features described in patients with *FIG4* pathogenic variants (Nicholson et al., 2011).

Whole exome sequencing for patient #2 revealed the presence of the c.364C>G (Leu122Val) *LITAF* gene variant in heterozygous state. Variants in the *LITAF* gene (stands for lipopolysaccharide‐induced tumor necrosis factor‐alpha factor, alternatively named *SIMPLE*) have been shown to be the cause of CMT type 1C (Street et al., 2003). The variant of our patient (c.364C>G, Leu122Val) has already been described as pathogenic by Saifi et al. (2005), as it has been found in heterozygous state in a patient with CMT type 1C. This patient, a 7‐year‐old girl, had shown mild distal weakness, pes cavus bilaterally, normal tendon reflexes, no sensory disturbances and, on nerve biopsy, chronic demyelination and remyelination presenting as onion bulbs. Testing of the non‐affected mother showed that she did not harbor the mutation; the father had died and was unavailable to testing (Saifi et al., 2005). The pathogenicity of the variant is also supported by multiple lines of computational evidence (CADD score of 25.5, Table 1).

The protein encoded by the *LITAF* gene is an endosomal transmembrane protein that is highly expressed in myelinating Schwann cells (Lee et al., 2013). The p.Leu122Val pathogenic variant found in our patient is located in the *LITAF* hydrophobic region, which is proposed to act as the transmembrane domain of the protein (Qin, Wunderley, Barrett, High, & Woodman, 2016). Supportive of the pathogenicity of this variant is the observation that all the *LITAF* pathogenic variants described so far are located either inside or in the vicinity of this hydrophobic transmembrane domain (Ciotti et al., 2014; Gerding, Koetting, Epplen, & Neusch, 2009; Lee, Olzmann, Chin, & Li, 2011; Qin et al., 2016; Saifi et al., 2005; Street et al., 2003; Werheid et al., 2016). Another argument in favor of the pathogenicity of the c.364C>G (Leu122Val) variant was the observation that it is highly conserved across evolution (Saifi et al., 2005). However, using COS cell cultures, Zhu et al. (2013) showed that the amount of the protein encoded by *LITAF* excreted in exosomes was not decreased as in the case of other pathogenic *LITAF* gene variants. The authors interpreted this as an indication that the c.364C>G (Leu122Val) *LITAF* change was a benign polymorphism. This was later counterargued by Ferreira Lacerda et al. (2014) who showed that this p.Leu122Val variant had an effect on the function of the *LITAF* protein product by leading to partial *LITAF* mislocalization. Specifically, the p.Leu122Val protein was found both in late endosomes/lysosomes and mitochondria and not exclusively in late endosomes/lysosomes as the wild type protein (Ferreira Lacerda et al., 2014). Of note, other *LITAF* pathogenic variants were completely mislocalized in mitochondria. Consistent with the results from the cell expression experiments described above, there is phenotypic variability in CMT patients harboring *LITAF* pathogenic variants, as these patients present either as CMT‐type phenotype or a predominantly sensory form (Guimarães‐Costa et al., 2017).

In patient #3, WES identified the hemizygous c.491G>A (p.Arg164Gln) known pathogenic variant in *GJB1* (OMIM 304040). This genetic variant has been previously reported in patients affected with the X‐linked dominant CMT neuropathy (CMT X1) (Bone, Deschênes, Balice‐Gordon, Fischbeck, & Scherer, 1997; Bort et al., 1997; Dubourg et al., 2001; Hoyer et al., 2014; Huehne et al., 2003; Kim et al., 2010; Mersiyanova et al., 2000; Panas, Karadimas, Avramopoulos, & Vassilopoulos, 1998; Tsai et al., 2016; Yoshihara et al., 2000), and appears to be a common pathogenic mutation in Greece (Karadima, Floroskufi, Koutsis, Vassilopoulos, & Panas, 2011; Koutsis et al., 2018).

Charcot–Marie–Tooth X1 caused by pathogenic variants in *GJB1* is the second most common subtype of CMT (Braathen, 2012; Dubourg et al., 2001; Huehne et al., 2003; Karadima et al., 2011; Li et al., 2016; Wang et al., 2015; Yoshihara et al., 2000), after CMT 1A which is caused by duplications in the *PMP22* gene. In large series of patients, the frequency of CMT X1 among diagnosed cases of CMT ranges from 5.9% to up to 10.7% (Braathen, 2012; DiVincenzo et al., 2014; Dohrn et al., 2017; Wang et al., 2015; Yuan et al., 2018). Since the first report of *GJB1* changes as a cause of CMT X1 in 1993 (Bergoffen et al., 1993), there have been over 450 pathogenic variants described in this gene (Bortolozzi, 2018).

Connexin 32, the protein encoded by *GJB1*, is an abundant protein in liver, but also found in the central and peripheral nervous system (Bortolozzi, 2018). It has been shown that it forms the membrane spanning hemichannel of gap junctions between adjacent cells. These gap junctions allow intercellular communication that is essential for cellular metabolism and growth (Bone et al., 1997). In the peripheral nervous system, it is found in the myelinating Schwann cells and localizes in the paranodal region (Bortolozzi, 2018); however, its exact cellular role has not been delineated yet. The c.491G>A sequence change found in our patient results in a substitution of arginine with glutamine at codon 164 (p.Arg164Gln) of the GJB1 protein. This protein spans the plasma membrane four times and the p.Arg164Gln change is located in the second extracellular loop (Tsai et al., 2016). According to this study by Tsai et al., the p.Arg164Gln change has an impact on the biophysical functions of GJB1‐encoded protein, and in particular leads to an impairment of channel ionic permeability. The arginine residue at codon 164 is highly conserved and it appears to be important for GJB1 protein function, since another missense variant, namely p.Arg164Trp, is also reported as pathogenic (Bort et al., 1997; Ionasescu, Searby, Ionasescu, & Meschino, 1995; Li et al., 2016; Soo Hyun et al., 2016; Wang et al., 2004).

MRI of the brain in our patient #3 showed a few small foci of abnormal signal intensity on T2‐weighted imaging in the subcortical white matter of the cerebral hemispheres. It has been shown that many patients affected by CMT X1 display such type of lesions in the MRI and even develop multiple sclerosis (Basri et al., 2007; Karadima et al., 2014; Koutsis et al., 2018; Koutsis, Karadima, Floroskoufi, Raftopoulou, & Panas, 2015; Yuan et al., 2018), or acute disseminated encephalomyelitis (Kim, Han, & Kim, 2017). This has been attributed to the presence of connexin 32 both in Schwann cells and in oligodendrocytes, although the exact pathophysiological mechanism underlying this CNS involvement is not completely elucidated (Kleopa & Scherer, 2006; Kleopa, Yum, & Scherer, 2002). It is of note that other patients described in the literature and harboring the same variant as our patient (p.Arg164Gln), had shown CNS involvement (Koutsis et al., 2018; Panas et al., 1998).

Here we have described three patients with CMT 4J, 1C, and X1, respectively, who have been diagnosed by WES after several years of nondiagnostic testing (both genetic and nongenetic), sparing them from their diagnostic and therapeutic uncertainty. These three cases exemplify the usefulness and practicality of a simplified approach, going directly from *PMP22* deletion/duplication testing to WES in diagnosing patients with hereditary polyneuropathies. Given the overlapping clinical and electrophysiological phenotypes of CMT disease caused by different gene defects and the increasingly low cost of WES compared to traditional Sanger sequencing, it is not anymore recommended to resort to sequential sequencing of various genes (Pipis, Rossor, Laura, & Reilly, 2019). Nowadays, the novel diagnostic techniques, and specifically WES, have the power to reveal the cause of rare CMT subtypes, ending the diagnostic odyssey of the patients, and preventing unnecessary and potentially harmful treatments.

## CONFLICT OF INTEREST

The authors have no conflict of interest to disclose.

## Data Availability

The data that support the findings of this study are available on request from the corresponding author. The data are not publicly available due to privacy or ethical restrictions.
