# Supplementary material for: Whole exome sequencing establishes diagnosis of Charcot–Marie–Tooth 4J, 1C, and X1 subtypes
Source: Mol Genet Genomic Med. 2020 Feb 5;8(4):e1141. doi: 10.1002/mgg3.1141 (PMC7196464; doi:10.1002/mgg3.1141)
Supplement: Supplementary file 4 [file MGG3-8-e1141-s004.docx]

**Supplementary Material**

Title:

Whole exome sequencing establishes diagnosis of Charcot-Marie-Tooth 4J, 1C and X1 subtypes

Kleita Michaelidou^1^, Ioannis Tsiverdis ^2^, Sophia Erimaki ^3^, Dimitra Papadimitriou ^4^, Georgios Amoiridis ^3^, Alexandros Papadimitriou ^4^, Panayiotis Mitsias^1,2,3,5^, Ioannis Zaganas ^1,2^

^1^Neurogenetics Laboratory, Medical School, University of Crete, Heraklion, Crete, Greece

^2^Neurology Department, University Hospital of Crete, Heraklion, Crete, Greece

^3^Neurophysiology Unit, University Hospital of Crete, Heraklion, Crete, Greece

^4^Henry Dunant Hospital Center and Biomedical Research Foundation Academy, Athens, Greece

^5^Department of Neurology, Henry Ford Hospital/Wayne State University, Detroit, Michigan, USA

**Supplementary Table 1.** Neurophysiological examination of patient #1.

**Supplementary Table 1A.** Nerve conduction studies of patient #1.

| Nerve stimulated | Stimulation site | Recording site | Distal latency (ms) | Amplitude* | Conduction velocity (m/s) | F-wave latency  (ms) |
| --- | --- | --- | --- | --- | --- | --- |
| Peroneal (m) L | Ankle  Below fibula  Above fibula | EDB | NR | NR | NR | NR |
| Tibial (m) L | Ankle  Popliteal fossa | AH | NR | NR | NR | NR |
| Median (m) L | Wrist  Antecubital fossa | APB | **6.1** | 4.0  **0.5** | **25.0** | **64.8**  **(Α-waves)** |
| Ulnar (m) L | Wrist  Below elbow  Above elbow | ADM | **4.4** | **2.5**  **1.2**  **1.0** | **20.2**  **22.0** | **64.8**  **(Α-waves)** |
| Median (m) R | Wrist  Antecubital fossa | APB | **8.3** | 3.2  **1.8** | **17.4** | NR |
| Ulnar (m)R | Wrist  Below elbow  Above elbow | ADM | **6.0** | 5.0  **2.6**  **NR** | **21.8**  **NR** | **61.8**  **(Α-waves)** |
| Sural (s) L | Calf | Lateral ankle | NR | NR | NR |  |
| Median (s) L | Index finger | Wrist | NR | NR | NR |  |
| Ulnar (s) L | Little finger | Wrist | NR | NR | NR |  |

**Supplementary Table 1B.** Electromyographic studies of patient #1.

| Muscle | Spontaneous activity | | | Voluntary motor unit action potentials | | | |
| --- | --- | --- | --- | --- | --- | --- | --- |
|  | Insertional activity | Fibrillation potentials | Fasciculations | Activation | Recruitment | Amplitude | polyphasia |
| Tibialis anterior L | NL | ++ | 0 | NL | ↓↓↓ | ↑↑ | 0 |
| Tibialis anterior R | NL | ++ | 0 | NL | ↓↓↓ | ↑↑ | 0 |
| Gastrocnemius L | NL | 0 | 0 | NL | ↓↓↓ | ↑ | 0 |
| Gastrocnemius R | NL | 0 | 0 | NL | ↓↓↓ | ↑↑ | 0 |
| Rectus Femoris L | NL | 0 | 0 | NL | ↓↓ | ↑ | 0 |
| Rectus Femoris R | NL | 0 | 0 | NL | ↓ | ↑ | 0 |
| Extensor digitorum communis L | NL | 0 | 0 | NL | ↓↓ | ↑ | 0 |
| Deltoid L | NL | 0 | 0 | NL | ↓ | NL | 0 |

Amplitude: motor in mV, sensory in μV.

The F-wave latencies represent the minimal F-wave latencies. Note the significantly reduced conduction velocities, the increased distal latencies, the presence of conduction block during proximal stimulation and the significant prolongation of F-wave latencies with the presence of A-waves.

***Abbreviations***

m: motor study, s: sensory study; L: left, R: right; EDB: extensor digitorum brevis, AH: abductor hallucis, APB: abductor pollicis brevis, ADM: abductor digiti minimi. NR: no response.

NL: normal, ↓ slightly decreased, ↓↓ moderately decreased, ↓↓↓ severely decreased, ↑ slightly increased, ↑↑ moderately increased, ↑↑↑ largely increased, 0 no change

**Supplementary Table2.** Neurophysiological examination of patient # 2.

**Supplementary Table 2A.** Nerve conduction studies of patient #2.

| Nerve stimulated | Stimulation site | Recording site | Distal latency (ms) | Amplitude | Conduction velocity (m/s) | F-wave latency  (ms) |
| --- | --- | --- | --- | --- | --- | --- |
| Peroneal (m) L, R | Ankle  Below fibula  Above fibula | EDB | NR | NR | NR | NR |
| Tibial (m)  L, R | Ankle  Popliteal fossa | AH | NR | NR | NR | NR |
| Median (m) L | Wrist  Antecubital fossa | APB | **4.8** | 6.3  **2.6** | **31.8** | **41.0** |
| Ulnar (m) L | Wrist  Below elbow  Above elbow | ADM | **3.5** | **1.7**  **1.0**  **1.0** | **26.4**  **25.0** | **51.0** |
| Median (m) R | Wrist  Antecubital fossa | APB | **4.4** | 5.9  **2.0** | **30.7** | **43.4** |
| Ulnar (m) R | Wrist  Below elbow  Above elbow | ADM | 2.5 | 6.1  **1.2**  **0.2** | **25.0**  **23.8** | **NR** |
| Sural (s) L | Calf | Lateral ankle | NR | NR | NR |  |
| Sural (s) R | Calf | Lateral ankle | NR | NR | NR |  |
| Median (s) L | Index finger | Wrist |  | **4.6** | **41.2** |  |
| Ulnar (s) L | Little finger | Wrist |  | **3.0** | **38.0** |  |

**Supplementary Table 2B.** Electromyographic studies of patient #2.

| Muscle | Spontaneous activity | | | Voluntary motor unit action potentials | | | |
| --- | --- | --- | --- | --- | --- | --- | --- |
|  | Insertional activity | Fibrillation potentials | Fasciculations | Activation | Recruitment | Amplitude | polyphasia |
| Tibialis anterior L | NL | 0 | 0 | NL | ↓ | ↑↑ | 0 |
| Peroneus brevis R | NL | 0 | 0 | NL | ↓↓↓ | ↑↑↑ | 0 |
| 1^st^ dorsal interosseus L | NL | 0 | 0 | NL | ↓↓ | ↑↑ | 0 |

Abbreviations as in **Supplementary Table 1**.

The sensory conduction velocities were measured using onset latencies. Note the mildly reduced conduction velocities with the presence of conduction block during proximal stimulation and the significant prolongation of F-waves.

**Supplementary Table3.** Neurophysiological examination of patient # 3.

**Supplementary Table 3A.** Nerve conduction studies of patient #3.

| Nerve stimulated | Stimulation site | Recording site | Distal latency (ms) | Amplitude | Conduction velocity (m/s) | F-wave latency  (ms) |
| --- | --- | --- | --- | --- | --- | --- |
| Peroneal (m) L, R | Ankle  Below fibula  Above fibula | EDB | NR | NR | NR | NR |
| Tibial (m)  L | Ankle  Popliteal fossa | AH | **8.0** | NR | **33** | NR |
| Tibial (m)  R | Ankle  Popliteal fossa | AH | **8.8** | NR | **36** | NR |
| Median (m) L | Wrist  Antecubital fossa | APB | **4.7** | NR | **37** | NR |
| Ulnar (m) L | Wrist  Below elbow  Above elbow | ADM | 3.7 | NR | **37** | NR |
| Median (m) R | Wrist  Antecubital fossa | APB | **4.6** | NR | **38** | NR |
| Ulnar (m) R | Wrist  Below elbow  Above elbow | ADM | **5.0** | NR | **33** | NR |
| Sural (s) L | Calf | Lateral ankle | - | NR | - | NR |
| Sural (s) R | Calf | Lateral ankle | - | NR | - | NR |
| Median (s) L | Index finger | Wrist | **5.0** | NR | NR | NR |
| Ulnar (s) L | Little finger | Wrist | **6.0** | NR | NR | NR |
| Median (s) R | Index finger | Wrist | **4.3** | NR | NR | NR |
| Ulnar (s) R | Little finger | Wrist | **7.0** | NR | NR | NR |

**Supplementary Table 3B.** Electromyographic studies of patient #3.

| Muscle | Spontaneous activity | | | Voluntary motor unit action potentials | | | |
| --- | --- | --- | --- | --- | --- | --- | --- |
|  | Insertional activity | Fibrillation potentials | Fasciculations | Activation | Recruitment | Amplitude | polyphasia |
| Tibialis anterior L | NL | + | NR | NL | ↓↓ | ↑↑ | ++ |
| Tibialis anterior R | NL | + | NR | NL | ↓↓ | ↑↑ | ++ |
| Extensor digitorum brevis L | NL | NR | NR | NL | ↓↓↓ | ↑↑↑ | 0 |
| Extensor digitorum brevis R | NR | NR | NR | No voluntary activity | | | |
| Flexor hallucis brevis L | NL | + | + | NL | ↓↓↓ | ↑↑↑ | 0 |
| Flexor hallucis brevis R | NL | + | + | NL | ↓↓↓ | ↑↑↑ | 0 |
| Abductor pollicis brevis L | NL | NR | NR | NL | ↓↓↓ | ↑↑↑ | + |
| Abductor pollicis brevis R | NL | NR | NR | NL | ↓↓↓ | ↑↑↑ | + |
| Abductor digiti minimi L | NL | NR | NR | NL | ↓↓↓ | ↑↑↑ | + |
| Abductor digiti minimi R | NL | NR | + | NL | ↓↓↓ | ↑↑↑ | + |

Abbreviations as in **Supplementary Table 1**.
